# Operation for Trachoma

**Published:** 1894-08

**Authors:** Thomas S. Mitchell

**Affiliations:** 1241½ Broad Street; Columbus, Ga.


### OPERATION FOR TRACHOMA.

By THOMAS S. MITCHELL, M.D., COLUMBUS, GA.

In reporting this case, I do not propose to add anything new to the already voluminous writings on the pathology, etiology and treatment of conjunctivitis granulosa, if possible, the most formidable disease the ophthalmic surgeon is called upon to treat, but to call attention to the fact that in suitable cases we have in Dr. Knapp's improved roller forceps a means by which a radical cure can be effected in a shorter time than by any other means now known to the profession.

On March 26, 1894, Minnie G., age five years, a healthy, redfaced child, was brought for treatment. She had had "sore eyes" for three years, and "everything in the world" had been tried with no avail. An examination revealed a well developed case of trachoma, the granulations being limited to the palpebral conjunctivæ and oculo-palpebral folds above, with considerable corneal involvement, especially of right eye, with its accustomed symptoms of lachrymation, photophobia, blepharospasm, etc. Dr. J. H. Shorter, of Macon, Ga., happened to be in my office at the time, and seeing the patient with me, concurred in my diagnosis and agreed that the case was a suitable one for expression.

The child's parents being averse to operative procedure, the following line of treatment was decided upon:

℞ Atropiæ sulphatis..........gr. ss.

Acidi borici..........gr. x.

Morphiæ sulphatis..........gr. ij.

Aquæ ditillatæ..........f℥ i.

M. Et sig. Two drops in each eye three to five times daily.

℞ Hydrig. oxid. flar..........gr. ij.

Petrolati..........ʒ ij.

M. Ft. ung. et sig. A small piece rubbed in conjunctival sac once daily.

In conjunction with this, patient was ordered brought to office daily to have lids "touched" with cupric sulphate. This latter procedure did not prove feasible, because patient was fractious and rebelled so at being brought to the office that the application was discontinued after one or two visits. Consent of the parents having been gotten, it was decided to operate by expressing the trachomatousbodies.

On April 2d, under chloroform (Squibbs), anesthesia administered by Dr. C. D. Wall, I expressed the granulations by placing one roller of the forceps far back in the superior fornix, while the other was placed on the conjunctival side of the tarsal cartilage, the lid having been everted and surface of granulations scarified with Graefe's linear cataract knife. Very slight hemorrhage followed the little operation, not more than a drachm from both eyes, and little or no reactionary inflammation. April 5th, patient convalescing satisfactorily; drops and ointment continued. May 1st, improvement continues—very little photophobia or pain. June 1st, patient's lids present an almost absolutely normal appearance; pain, lachrymation and photophobia have disappeared, and child plays in sunlight with the eyes unprotected and does not complain; corneal involvement gone, leaving in its wake an opacity in the upper and outer quadrant of right eye extending near the pupil, for which I have dusted in the eye once daily boric acid and papoid, equal parts, as advocated by Dr. J. A. Lydston, of Chicago, at the meeting of the A. M. A., 1893, with the gratifying results of seeing the opacity grow clearer.

At this writing, July 13th, the patient continues free from symptoms and the opacity continues to clear up. If I have succeeded in calling attention to the importance of operating by expression for the removal of trachoma bodies, the object of this paper will not have been defeated.

1241½ *Broad Street.*

